# The potential synergistic effects between psychedelic administration and nature contact for the improvement of mental health

**DOI:** 10.1177/2055102920978123

**Published:** 2020-12-06

**Authors:** Sam Gandy, Matthias Forstmann, Robin Lester Carhart-Harris, Christopher Timmermann, David Luke, Rosalind Watts

**Affiliations:** 1 Centre for Psychedelic Research, Imperial College London, UK; 2 Synthesis Institute, The Netherlands; 3 University of Zurich, Switzerland; 4 University of Greenwich, UK

**Keywords:** drug effects, health promotion, health psychology, well-being, psychedelics

## Abstract

Therapeutic psychedelic administration and contact with nature have been associated with the same psychological mechanisms: decreased rumination and negative affect, enhanced psychological connectedness and mindfulness-related capacities, and heightened states of awe and transcendent experiences, all processes linked to improvements in mental health amongst clinical and healthy populations. Nature-based settings can have inherently psychologically soothing properties which may complement all stages of psychedelic therapy (mainly preparation and integration) whilst potentiating increases in nature relatedness, with associated psychological benefits. Maximising enhancement of nature relatedness through therapeutic psychedelic administration may constitute an independent and complementary pathway towards improvements in mental health that can be elicited by psychedelics.

## Introduction

Nature relatedness has been associated with a broad range of benefits to psychological health and well-being. It is a measurable, trait-like construct of one’s self-identification with nature, defined as a sense of ‘oneness with the natural world’ (Mayer and Frantz, 2004) or a ‘sustained awareness of the interrelatedness between one’s self and the rest of nature’ (Zylstra et al., 2014). It is a stable state of consciousness, that is experiential, emotional and highly personal, rather than rational or deliberation-based (Lumber et al., 2017; Nisbet et al., 2009; Nisbet and Zelenski, 2013; Richardson and Sheffield, 2017; Wright and Matthews, 2015; Zylstra et al., 2014). Nature relatedness is considered to be a basic psychological human need (Baxter and Pelletier, 2019), associated with mental well-being and also with increased contact with nature (Fretwell and Greig, 2019; Lin et al., 2014; Mayer and Frantz, 2004; Nisbet et al., 2009, 2011; Nisbet and Zelenski, 2013; Tam, 2013; Van Gordon et al., 2018; Wolsko and Lindberg, 2013; Wright and Matthews, 2015). Contact with nature is associated with an extraordinarily broad range of benefits to physical and mental health and well-being (for reviews see Frumkin et al., 2017; Twohig-Bennett and Jones, 2018). Nature relatedness (sometimes referred to as nature connectedness in the literature) is distinct from nature contact, yielding independent and additive benefits, although the two have a positively reinforcing relationship.

Research suggests that experiences with classical or serotonergic psychedelic compounds (acting as agonists at the serotonin 5-HT_2A_ receptor) (Carhart-Harris et al., 2014; Glennon et al., 1984; Nichols, 2016) can foster sustained increases in nature relatedness (Forstmann and Sagioglou, 2017; Kettner et al., 2019; Lyons and Carhart-Harris, 2018) and appreciation for and contact with nature (Doblin, 1991; Luke, 2017; Kangaslampi et al., 2020; Noorani et al., 2018; Studerus et al., 2011; Watts et al., 2017). Furthermore, access to nature-based settings during psychedelic sessions predicts positive changes in nature relatedness (Kettner et al., 2019). An increase in nature relatedness likely occurs through a number of different mechanisms, such as through increased mindfulness-related capacities, connectedness, openness to experience and eliciting strong emotional states (for a review see Aday et al., 2020). That psychedelics such as psilocybin are capable of eliciting sustained increases in nature relatedness even when administered in clinical settings lacking in nature (Lyons and Carhart-Harris, 2018) is a noteworthy finding.

Increased nature relatedness is just one of many psychological benefits that can be occasioned through therapeutic administration of psychedelics to both healthy and clinical populations. In clinical populations, psychedelic substances are currently being investigated for the treatment of major depressive disorder and existential anxiety secondary to a terminal cancer diagnosis (Agin-Liebes et al., 2020; Barrett et al., 2020; Carhart-Harris et al., 2016a, 2018a; Gasser et al., 2014; Griffiths et al., 2016; Grob et al., 2011; Ross et al., 2016), addiction (Bogenschutz et al., 2015; Bogenschutz and Johnson, 2016; Johnson et al., 2014, 2016) and PTSD (Krediet et al., 2020). For reviews of clinical studies with psychedelics see dos Santos et al. (2018). In studies looking at psychedelic administration amongst healthy populations, sustained increases across numerous measures of psychological well-being are observed (see reviews by Aday et al., 2020; Gandy, 2019).

The mechanisms by which psychedelics confer benefits to well-being share overlap with how nature relatedness and nature contact yield benefits. Psychedelics have been associated with eliciting sustained increases in mindfulness-related capacities (Madsen et al., 2020; Murphy-Beiner and Soar, 2020; Sampedro et al., 2017; Soler et al., 2018; Uthaug et al., 2019), facilitating enhanced connectedness, empathy and unitive states (Carhart-Harris et al., 2018b; Forstmann et al., 2020; Griffiths et al., 2018; Mason et al., 2019; Noorani et al., 2018; Pokorny et al., 2017; van Mulukom et al., 2020; Watts et al., 2017) and eliciting awe (Griffiths et al., 2006; Hendricks, 2018; Noorani et al., 2018; Richards et al., 1977; Watts et al., 2017). Psychedelics have also been found to increase openness to experience in an enduring way (Barrett et al., 2020; Erritzoe et al., 2018; Lebedev et al., 2016; MacLean et al., 2011; Madsen et al., 2020).

In this review, we lay out the argument for utilising nature-based settings and practices for some stages of psychedelic therapy (mainly preparation and integration). The current model for psychedelic therapy requires the psychedelic sessions themselves take place in a secure clinical setting for the safety and predictability this provides. However, it may be that incorporating some elements of nature contact and connection into the preparation and integration phases could support the therapy model, potentially by amplifying some of the key therapeutic mechanisms, whilst also increasing the likelihood that a patient will use nature as an ongoing resource. The potential of psychedelics to increase nature relatedness may warrant the development of a new model of psychedelic therapy specifically focused on increasing nature relatedness for the sake of the well-being this can bring to the individual, and the nature-protective potential this could bring to our communities. Such a model could be used with a healthy (non-clinical) population, with whom the possibility of conducting the psychedelic session itself in a secure and sheltered natural environment could be explored.

## Mental health benefits of nature relatedness and contact

### Nature contact and mental health

The inherent healing power of nature has been recognised for centuries (Olmsted, 1865; Kempf, 1905; Ward Thompson, 2011). Nature can be defined in this context as ‘areas containing elements of living systems that include plants and nonhuman animals across a range of scales and degrees of human management, from a small urban park through to relatively “pristine wilderness”’ (Bratman et al., 2012: 120).

When it comes to mental health, contact with nature is associated with reductions in mental distress (White et al., 2013), anxiety (Beyer et al., 2014; Bratman et al., 2015a; Maas et al., 2009; Nutsford et al., 2013; Song et al., 2013, 2015) and depression (Astell-Burt et al., 2014; Berman et al., 2012; Beyer et al., 2014; Cohen-Cline et al., 2015; Gascon et al., 2015; Maas et al., 2009; McEachan et al., 2016; Nutsford et al., 2013; Shanahan et al., 2016). Nature contact can yield improvements in mood and memory in patients suffering from major depressive disorder (Berman et al., 2012) and improve symptoms of post-traumatic stress disorder (PTSD) (Anderson et al., 2018a, 2018b; Poulsen, 2017; Poulsen et al., 2016). It can also reduce rumination (Bratman et al., 2015a, 2015b), and stress levels (for reviews see Berto, 2014; Haluza et al., 2014), with this stress reduction being an important health benefit alone and a potential mechanism for further health benefits (Lovallo, 2015).

Contact with nature is a strong predictor of psychological well-being (Bowler et al., 2010; Capaldi et al., 2015; McMahan and Estes, 2015), elevating both hedonic and eudaimonic well-being (Capaldi et al., 2015) and facilitating psychological restoration (Berman et al., 2008; Hartig, 2008; Hartig et al., 1991, 2011; Korpela et al., 2014; van den Berg et al., 2014), the latter being defined as the renewal of psychological resources depleted by mental exertion or stress, such as attention and mood (Hartig et al., 2001). Time in nature has been found to increase vitality (Ryan et al., 2010), self-esteem (Barton and Pretty, 2010), and result in higher positive affect (associated with the extent a person feels happy, joyous, interested and active) and lower negative affect (associated with a predisposition towards negative feelings such as emotional distress and states such as sadness, guilt and fear) (Bratman et al., 2015a; MacKerron and Mourato, 2013; McMahan and Estes, 2015; Neill et al., 2019; Passmore and Holder, 2017; van den Bosch and Sang, 2017). Even 5–10 minutes spent in a natural setting is sufficient to improve psychological well-being and lower anxiety and stress levels (Meredith et al., 2020; Neill et al., 2019).

### Nature relatedness and mental health

In addition to nature contact, there is a substantial body of research literature that highlights a strong association between nature *relatedness* and psychological health and eudaimonic well-being, that is, subjective experiences linked to living a life of virtue in pursuit of human excellence, associated with experiences of self-actualisation, vitality and personal expressiveness (for a review see Pritchard et al., 2020). One study reported a positive relationship between nature relatedness and eudaimonic well-being that was nearly four times larger than the increase in the latter associated with higher socio-economic status (Martin et al., 2020).

Specifically, nature relatedness has been associated with enhanced vitality (Capaldi et al., 2014; Cervinka et al., 2012; Ryan and Frederick, 1997), greater perceived life meaning (Cervinka et al., 2012; Nisbet et al., 2011), life satisfaction (Mayer and Frantz, 2004), feelings of worthwhileness (Fretwell and Greig, 2019; Martin et al., 2020), greater happiness and positive affect (Capaldi et al., 2014; Fretwell and Greig, 2019; Mayer et al., 2009; Nisbet et al., 2011; Pritchard et al., 2020; Zelenski and Nisbet, 2014) and reduced levels of anxiety (Capaldi et al., 2014; Martyn and Brymer, 2016; Zelenski and Nisbet, 2014). In addition, nature relatedness is associated with higher levels of self-reported personal growth (Pritchard et al., 2020), enhanced psychological functioning (Sobko et al., 2018) and resilience (Ingulli and Lindbloom, 2013). Nature relatedness is also associated with physical activity (Puhakka et al., 2018), which is linked to good mental health (Saxena et al., 2005; Taylor et al., 1985). While nature relatedness is associated with a broad range of psychological benefits, it may be associated with modern health worries perceived to result from living in artificial environments (Dömötör et al., 2017).

Nature relatedness is also a strong predictor of pro-environmental attitudes and behaviour (Diessner et al., 2018; Dutcher et al., 2007; Forstmann and Sagioglou, 2017; Geng et al., 2015; Gkargkavouzi et al., 2019; Mackay and Schmitt, 2019; Martin et al., 2020; Mayer and Frantz, 2004; Nisbet et al., 2009; Nisbet and Zelenski, 2013; Otto and Pensini, 2017; Restall and Conrad, 2015; Richardson et al., 2020; Tam, 2013; Whitburn et al., 2020; Zylstra et al., 2014), this being a salient finding, given a notable lack of effective interventions for reversing environmentally damaging human behaviour (Prescott and Logan, 2017). Pro-environmental behaviour has been associated with well-being (Corral-Verdugo et al., 2013; Kaida and Kaida, 2016; Netuveli and Watts, 2020; Prati et al., 2017) and strongly linked to prosociality (the intent to benefit others) – with the two likely being mutually reinforcing (Neaman et al., 2018). However, aspects of nature relatedness reflecting self-identification with nature and a conservation worldview may be associated with increased depression, anxiety or stress (Dean et al., 2018), likely due to increased awareness of human driven ecological damage and destruction.

Nature relatedness acts as an important mediator for some of the benefits obtained while spending time in nature. For example, nature relatedness was reported to mediate the link between contact with nature and subjective well-being and ecological behaviour (Martin et al., 2020; Pensini et al., 2016) in addition to mediating the relationship between engagement with natural beauty and pro-environmental behaviour (Diessner et al., 2018). Likewise, it was found to partially mediate a number of other positive effects experienced in natural settings such as increased attentional capacity, propensity to experience positive emotions and the ability to reflect on a life problem (McMahan et al., 2018; Mayer et al., 2009), while promoting a more positive body image (Swami et al., 2016, 2020). Furthermore, nature relatedness was found to be positively associated with the perceived restorativeness of natural settings (Berto et al., 2018), psychological benefits obtained from outdoor exercise (Lawton et al., 2017; Loureiro and Veloso, 2014) and predicts life satisfaction and self-esteem when individuals are attuned to nature’s beauty (Zhang et al., 2014). Following contact with nature, higher levels of nature relatedness have been found to positively predict life satisfaction (Chang et al., 2020), transcendent and awe-inspiring experiences (Davis and Gatersleben, 2013) and to elicit higher valuations of intrinsic (e.g. personal growth, intimacy and community) as opposed to extrinsic (e.g. money, image and social status) aspirations (Dopko, 2017; Weinstein et al., 2009).

Research indicates that childhood is a crucial life period for the development of a bond with nature (Berk, 2006; Chawla, 1999; Kals et al., 1999; Kellert, 2002; Ward Thompson et al., 2008). Greater contact with nature during childhood is associated with greater nature relatedness, contact with nature and pro-environmental behaviours in adulthood (Chawla, 1999; Chawla and Cushing, 2007; Chawla and Derr, 2012; Chawla and Flanders, 2007; Fretwell and Greig, 2019; Hinds and Sparks, 2008; Kals et al., 1999; Laird et al., 2014; Lohr and Pearson-Mims, 2005; Rosa et al., 2018; Tam, 2013; Wells and Lekies, 2006). However, as urbanisation increases globally (Dye, 2008; Eigenbrod et al., 2011; United Nations, 2018), increasing numbers of people are being brought up in nature-depleted environments (Eigenbrod et al., 2011; Soga and Gaston, 2016; Turner et al., 2004; van den Berg et al., 2007) which is likely to negatively impact people’s connection to nature (Fretwell and Greig, 2019). In addition, increasing usage of electronic entertainment technology and smartphones appears to be fuelling a growing disconnection from nature (Larson et al., 2018; Pergams and Zaradic, 2006; Richardson et al., 2018). This disconnection is further evidenced by a shift away from nature-based content in media and cultural products since the 1950’s (Kesebir and Kesebir, 2017; Prévot-Julliard et al., 2015).

There is a need for interventions able to foster sustained increases in nature relatedness (Frantz and Mayer, 2014; Nisbet and Zelenski, 2014; Richardson and Sheffield, 2017; Wright and Matthews, 2015), as passive contact with nature alone may only elicit transient increases (Nisbet and Zelenski, 2014) or be insufficient to increase it (Ernst and Theimer, 2011; Hamann and Ivtzan, 2016; Zylstra et al., 2014). Investigations into experimental manipulations of nature relatedness are lacking (Richardson and Sheffield, 2017). From what is known, with the exception of being an occasional consequence of passive and active nature contact, nature relatedness appears to be a deeply held and stable trait, and seems resistant to change like other environmental attitudes (Nisbet and Zelenski, 2014; Wright and Matthews, 2015) and personality traits (Terracciano et al., 2005, 2006).

## Potential beneficial synergy of psychedelics and nature contact

### Overlapping mechanisms between psychedelic administration and contact with nature

#### Neurobiological

Natural settings may elicit mind/brain states that share some similarities with psychedelic mind/brain states. Subjects walking in forests have been found to exhibit stronger functional connectivity between different brain regions than people walking in busy urban environments (Chen et al., 2015). Similarly, psychedelics appear to reduce modular activity, while enhancing global connectivity in the brain (Carhart-Harris et al., 2016c; Tagliazucchi et al., 2016), with effects extending at least a month after the psychedelic session (Barrett et al., 2020).

Both psychedelics (Barrett et al., 2020; Carhart-Harris et al., 2012; Speth et al., 2016; Watts et al., 2017) and contact with nature (Bratman et al., 2015a, 2015b) appear to reduce rumination and activity in areas of the brain implicated in depression, including the default mode network (DMN) (Hamilton et al., 2015). The DMN is involved with capacities involving self-projection, including remembering the past, envisioning the future and considering the thoughts and perspectives of others (Buckner and Caroll, 2007; Spreng and Grady, 2010) with parts of this brain region thought to mediate the sense of self (Letheby and Gerrans, 2017; Smigielski et al., 2019b). Rumination has been linked to the DMN (Zhou et al., 2020) and increased functional connectivity between the DMN and subgenual prefrontal cortex (sgPFC), including increased regional cerebral blood flow in the latter (Hamilton et al., 2015). Rumination is associated with mood disorders such as depression and anxiety (for a review see Olatunji et al., 2013) and is an important predictor and maintaining factor of persistent PTSD (for a review see Szabo et al., 2017). Contact with nature appears to reduce activity in the subgenual prefrontal cortex (sgPFC) which is a major node of the DMN (Bratman et al., 2015b). Similarly, intravenous psilocybin administration has been found to acutely decrease blood flow and metabolism in the sgPFC and also the posterior cingulate cortex (PCC) which is another major node of the DMN (Carhart-Harris et al., 2012).

#### Psychological

##### Connectedness

Connectedness has been suggested as a key phenomenon relevant to both the acute action of psychedelics and their longer-term effects (Carhart-Harris et al., 2018b; Watts et al., 2017). The construct of connectedness is currently being defined as an empathic and embodied sense of closeness to self, others and world/universe. An upcoming measure of connectedness, the Watts Connectedness Scale (WCS) has three subscales (i) ‘connection to self’ which includes connection to senses, emotions, values and life meaning; (ii) ‘connection to others’ which includes feeling part of the surrounding environment and empathy for others (iii) ‘connection to world/universe’ which includes connection with nature, the ‘bigger picture’ and feeling that everything is interconnected. All of these are important components of the capacity for communion with the natural world. We propose that the ability of psychedelics to increase nature relatedness may be a component of a more general sense of connectedness so often associated with the psychedelic experience.

Ego-dissolution and the (arguably mutually dependent) unitive experience may be central to the experiences of increased interconnectedness that can occur. Ego-dissolution has been described as ‘a disruption of ego-boundaries, which results in a blurring of the distinction between self-representation and object-representation’ (Nour et al., 2016) and is strongly associated with nature relatedness, both retrospectively (Nour et al., 2017) and prospectively (Kettner et al., 2019) suggesting this relationship is causative, rather than merely correlative. This dissolution of boundaries is reliably occasioned by psychedelics, and may result in feelings of unity and oneness with nature (Grob, 2002; Grof, 1980) and the universe (Riba et al., 2001). Additionally, administration of psilocybin has been found to elicit dose-dependent increases in measures of ‘external unity’ (Griffiths et al., 2006, 2008, 2011), or feelings of interconnectedness with the external world.

Nature contact itself can lead to greater connectedness, acting in a similar way to psychedelics. Just as ego-dissolution under psychedelics is associated with the dissolution of self-referential boundaries (Johnson et al., 2008), connectedness to nature can yield a similar effect (Dutcher et al., 2007). Nature relatedness is also associated with empathy (Metz, 2014), and an increased acknowledgement of nature has also been implicated in enhancing connectedness to other people and life as a whole (Passmore and Holder, 2017).

The capacity of psychedelics and nature contact to increase a sense of connectedness is notable, as a sense of ‘disconnection’, alienation or isolation has been implicated with a broad range of mental illnesses including eating disorders (Huemer et al., 2011), bipolar personality disorder (Kverme et al., 2019), PTSD (McDermott et al., 2012) and depression (Karp, 2017; Sorajjakool et al., 2008; Watts et al., 2017). Interestingly, feelings of disconnection from nature and other humans are not uncommon insights described by psychedelic users (St John, 2018), and this disconnection is frequently viewed as a source of health and societal problems, with these substances perceived to partly facilitate healing by amending this disconnection (Fotiou, 2012; Gearin, 2015, 2017; Schmid, 2013; St John, 2018; Watts et al., 2017; Winkelman, 2013). Connectedness is considered a key predictor and mediator of well-being (Capaldi et al., 2015; Cervinka et al., 2012; Lee et al., 2008; Saeri et al., 2018; Zelenski and Nisbet, 2014), in addition to a factor linked to recovery of mental health, including recovery from depression and addiction (Drake and Whitley, 2014; Leamy et al., 2011).

##### Mystical experience

Feelings of interconnectedness are a core facet of the peak or mystical-type experiences that psilocybin can occasion (Barrett and Griffiths, 2018; MacLean et al., 2011). Other core features of this experience include deep feelings of unity, a sense of sacredness, deeply felt positive mood, a sense of transcending time and space, ineffability and paradoxicality and a noetic quality (Griffiths et al., 2006). In addition to being a key component of the long-term benefits reported in both clinical and healthy populations undergoing psychedelic sessions (Aday et al., 2020; Barrett and Griffiths, 2018; Johnson et al., 2019), this experience has been found to be strongly associated with enduring positive changes in people’s relationship to nature in a retrospective study of people’s first psychedelic experiences (Kangaslampi et al., 2020). Inversely, nature-based settings appear prone to eliciting transcendent or mystical-type experiences (Ashley, 2007; Bethelmy and Corraliza, 2019; Harrild and Luke, 2020; Laski, 1961; Marshall, 2005; Snell and Simmonds, 2012; Williams and Harvey, 2001) which may include experiential components such as deeply felt positive mood, unity, timelessness and states of mindful absorption. In addition, one study comparing mystical experiences that occur in natural and human-built settings found that both significantly predicted psychological well-being, but only mystical experiences occurring in natural settings predicted an increase in pro-environmental behaviour (Snell and Simmonds, 2015). Mystical-type experiences occasioned by psychedelics are likely linked to the sustained increases in measures of spirituality reported by people that take them (Lerner and Lyvers, 2006; Móró et al., 2011; for a review see Aday et al., 2020). Spiritual feelings occur on deeper levels of experience than the intellect alone, involving emotions and meaning, and perceptions of connecting to something larger than oneself (Schroeder, 1992), similar to nature relatedness (Howell et al., 2011; Lumber et al., 2017).

##### Awe

The experience of awe has been linked to enhanced well-being (Anderson et al., 2018b; Dong and Ni, 2019; Rudd et al., 2012), life satisfaction (Rudd et al., 2012), prosociality (Bai et al., 2017; Piff et al., 2015; Sturm et al., 2020), and reduced negative affect (Lopes et al., 2020), and mental distress (Sturm et al., 2020), in addition to being associated with nature relatedness (Bethelmy and Corraliza, 2019) and pro-environmental behaviour (Wang and Liu, 2019; Zhao et al., 2018), all enduring effects associated with psychedelic use (Gandy, 2019). Psychedelics have been found to elicit feelings of awe (Griffiths et al., 2006; Hendricks, 2018; Noorani et al., 2018; Riba et al., 2001; Richards et al., 1977; Watts et al., 2017), and an enhancement of awe may persist beyond the acute experience (Noorani et al., 2018). This in turn has been linked to enhanced feelings of connectedness and empathy (Nelson-Coffey et al., 2019; van Mulukom et al., 2020). Nature can be considered a prototypical inducer of awe (Bethelmy and Corraliza, 2019; Keltner and Haidt, 2003), with experiences of awe more reliably triggered by exposure to natural rather than built environments (Ballew and Omoto, 2018). It has been proposed that administering psychedelics in natural settings known to elicit awe may enhance treatment efficacy of psychedelic therapy if safety is ensured (Hendricks, 2018). Experiences of awe in nature may be associated with perception of large natural objects such as mountains or vistas, events such as storms, or objects with infinite repetition, including waves and fractal patterns, such as trees, clouds, rain and birdsong – and a ‘smallness of self’ in this context (Forsythe and Sheehy, 2011; Keltner and Haidt, 2003; Richards, 2001; Shiota et al., 2007; Sturm et al., 2020). Perception of fractal patterns is also commonly associated with the visual imagery elicited by psychedelics (Klüver, 1966; Varley et al., 2020). Awe is deeply tied to feelings of spirituality (Hu et al., 2018; Kearns and Tyler, 2020; Preston and Shin, 2017; Van Cappellen and Saroglou, 2012), and spirituality and nature relatedness appear to be strongly linked (de Jager Meezenbroek et al., 2012; Dömötör et al., 2017; Saraglou et al., 2008; Trigwell et al., 2014). Spirituality can act as a mediator between nature relatedness and contact with nature and psychological well-being (Kamitsis and Francis, 2013; Knepple Carney and Patrick, 2016; Trigwell et al., 2014).

##### Reduction of negative affect

Psychedelics may elicit strong emotional states during the acute experience, and challenging emotions such as fear or grief are not uncommon (Belser et al., 2017; Griffiths et al., 2006, 2011; Haijen et al., 2018; Prepeliczay, 2002; Studerus et al., 2012). However, their longer term impact on affect has been consistently demonstrated to be positive. Reductions in negative affect are usually reported the day after a psychedelic session and tend to endure for weeks or months (Barrett et al., 2020; Carhart-Harris et al., 2016a, 2018a; Uthaug et al., 2018, 2019; Watts et al., 2017). Both nature relatedness and nature contact appear to have similar and potentially synergistic effects on reducing negative affect (Bratman et al., 2015a; Capaldi et al., 2014; Hamann and Ivtzan, 2016; Lopes et al., 2020; Mayer et al., 2009; McMahan and Estes, 2015; McMahan et al., 2018; Neill et al., 2019; Nisbet et al., 2011; Passmore and Holder, 2017; Pritchard et al., 2020; van den Bosch and Sang, 2017; Zelenski and Nisbet, 2014).

Major depression diagnoses are characterised by high levels of negative affect, with concurrent attenuated levels of positive affect (Boumparis et al., 2016; Clark and Watson, 1991; Watson et al., 1988; Watson and Naragon-Gainey, 2010), this being established in numerous studies (Brown et al., 1998; Kring et al., 2007; Lonigan et al., 2003). Negative affect has been found to act as general predictor of psychiatric disorder, and is strongly associated with mood and anxiety disorders (Hofmann et al., 2012), substance craving (Martel et al., 2014; Schlauch et al., 2013; Sinha and O’Malley, 1999; Volkow et al., 2016; Witkiewitz and Villarroel, 2009) and with rumination in depression (Iqbal and Dark, 2015; Thomsen, 2006). A reduction in negative affect has been found to reduce the strength and frequency of substance cravings following contact with natural environments (Martin et al., 2019).

##### Mindfulness

Mindfulness has been defined as ‘being attentive to and aware of what is taking place in the present’ (Brown and Ryan, 2003: 822). Psychedelics can foster enduring increases in measures of mindfulness-related capacities (Madsen et al., 2020; Murphy-Beiner and Soar, 2020; Sampedro et al., 2017; Soler et al., 2018; Uthaug et al., 2019), even when used outside the context of a mindfulness meditation practice. Cultivating mindfulness also enhances qualities of the acute psychedelic experience, in addition to the long-term psychological benefits obtained from psychedelic use (Griffiths et al., 2018; Smigielski et al., 2019a). Mindfulness has also been found to be related to both nature relatedness and psychological well-being (Howell et al., 2011) and is associated with pro-environmental behaviour (Barbaro and Pickett, 2016). There is a synergistic, positive association between mindfulness and nature relatedness (Andersen, 2017; Aspy and Proeve, 2017; Schutte and Malouff, 2018; Unsworth et al., 2016; Van Gordon et al., 2018), and the former has been found to enhance the latter in natural settings (Nisbet et al., 2019; Unsworth et al., 2016). Other studies confirm nature relatedness to be strongly associated with mindfulness (Howell et al., 2011; Wolsko and Lindberg, 2013).

Contact with natural settings can yield meditative, reflective mind states (Aspinall et al., 2015) and perception of fractals in nature may induce alpha activity in the brain, an indicator of a wakefully relaxed state and internalized attention (Hägerhäll et al., 2015). Nature contact can increase mindfulness (Hamann and Ivtzan, 2016; Richardson and Hallam, 2013; Van Gordon et al., 2018) and the benefits of mindfulness appear to be enhanced in nature-based settings (Araci, 2018; Choe et al., 2020; Gerard, 2018; Lymeus, 2019; Van Gordon et al., 2018). In turn, mindfulness can enhance the benefits yielded by nature-based settings (Van Gordon et al., 2018), acting as a mediator between nature contact and psychological well-being (Stewart and Haaga, 2018). Enhanced mindfulness capacities are associated with reductions in rumination (Jury and Jose, 2019; Williams, 2008), positive outcomes in the treatment of depression and addiction (Brewer et al., 2010; Deng et al., 2014; Williams, 2008; Witkiewitz and Bowen, 2010) and may contribute to the treatment of PTSD (Boyd et al., 2018; Hopwood and Schutte, 2017).

#### Personality

Psychedelics have been shown to increase personality trait openness to experience in an enduring way (Barrett et al., 2020; Carhart-Harris et al., 2016b; Erritzoe et al., 2018; Griffiths et al., 2018; Lebedev et al., 2016; MacLean et al., 2011; Madsen et al., 2020; Netzband et al., 2020). Openness is one of the primary personality correlates of connectedness to nature (Lee et al., 2015; Nisbet et al., 2009; Richardson and Sheffield, 2015) and pro-environmental behaviour (Markowitz et al., 2012; Puech et al., 2019; Wuertz, 2015), and predicts a propensity for awe-like experiences (Dong and Ni, 2019), including in response to nature (Silvia et al., 2015). It is associated with a number of traits including aesthetic appreciation (MacLean et al., 2011), one of the enduring effects linked to psilocybin usage (Noorani et al., 2018; Watts et al., 2017; Studerus et al., 2011). Appreciation of aesthetics in nature may partly explain the positive relationship between people and nature and act as a pathway to enhanced nature relatedness (Capaldi et al., 2017; Lumber et al., 2017; Zhang et al., 2014). Ayahuasca users have been found to rate more highly in the personality trait of self-transcendence (Bouso et al., 2012; Jiménez-Garrido et al., 2020), which is linked to an expansion of personal boundaries to encompass that which is greater than the self, and is strongly related to openness (De Fruyt et al., 2000). Like openness, it is also a predictor of nature relatedness and pro-environmental attitudes (Dornhoff et al., 2019; Tam, 2013).

Psilocybin was found to increase trait absorption (characterised by a disposition to become absorbed in one’s internal mental imagery) for at least a month post experience in healthy volunteers (Barrett et al., 2020). Absorption predicts a proclivity towards experiencing awe and positive emotional states in response to natural but not built settings (Ballew and Omoto, 2018; van Elk et al., 2016), in addition to predicting response to psilocybin (Russ et al., 2019; Studerus et al., 2012). Psilocybin therapy for treatment resistant depression was found to significantly reduce trait neuroticism scores (Erritzoe et al., 2018), with psilocybin administration among healthy volunteers also found to lower neuroticism (Barrett et al., 2020). In addition, ayahuasca usage in a traditional context was found to significantly reduce neuroticism, with changes sustained at 6-month follow up (Netzband et al., 2020). This is notable, as individuals reporting lower levels of neuroticism appear to gain greater psychological benefits through contact with nature (Ambrey and Cartlidge, 2017).

## How to maximise nature relatedness using psychedelics

### Potential benefits of natural settings for therapeutic psychedelic experiences

Classical psychedelics such as psilocybin are currently designated as Schedule 1 drugs in the UK and USA, imposing onerous and highly restrictive regulations around their use in a research and therapeutic context (Aday et al., 2020; Nutt et al., 2013), with clinicians calling on such restrictions to be revised to more fairly reflect their relative harm and potential benefit and to facilitate greater access for research and potential medical development (Johnson et al., 2018; Nutt et al., 2020; Rucker, 2015). Presently, most human psilocybin studies occur in monitored hospital or research settings, despite psilocybin in its naturally occurring fungal-form having an ancient history of human usage (Nichols, 2020). Psilocybin has a very favourable toxicity profile and negligible addiction potential and a number of independent analyses reporting that it has a benign safety profile (Carhart-Harris and Goodwin, 2017; Hasler et al., 2004; Johnson et al., 2018; Nutt et al., 2010; Rucker et al., 2019; van Amsterdam et al., 2011; Winstock et al., 2019).

The set (immediate and extended psychological context) and setting (extended sociocultural and immediate environmental context) framing psychedelic usage is known to be a key determinant of experiential outcomes (Carhart-Harris et al., 2018c; Eisner, 1997; Hartogsohn, 2016, 2017; Johnson et al., 2008; Leary et al., 1963; Masters and Houston, 1966). In clinical settings, psilocybin is often administered in a pre-prepared hospital room or living-room-like environment (Carhart-Harris et al., 2016a; Griffiths et al., 2006; Johnson et al., 2008; Krediet et al., 2020). People undergoing clinical psilocybin sessions typically wear eyeshades and headphones playing music, and they are instructed to focus their attention inwards (Johnson et al., 2008; Krediet et al., 2020) as an internal focus is required to limit distractions and facilitate the processing of autobiographical content that can arise (Grof, 1980; Schenberg, 2018). However, in an inwardly focused therapeutic session, where eyeshades are worn, there are many instances where the participant removes the eyeshades. For example, discussions with the therapists tend to take place with the eyeshades removed whereby the participant will be aware of the surrounding environment.

Part of the efficacy of psychedelics when utilised in a therapeutic context appears to be through their capacity to act as catalysts or amplifiers of psychotherapeutic practices and processes (Grof, 1980; Sloshower et al., 2020; Watts et al., 2017; Watts and Luoma, 2020). Nature seems to also act as an amplifier of therapeutic effect: one study found that cognitive behaviour therapy (CBT)-based psychotherapy applied in a forest environment was helpful in achievement of remission of major depression among sufferers, with the forest setting enhancing the effect of the psychotherapeutic intervention when compared to a clinical hospital setting (Kim et al., 2009). A course of forest therapy was also found to hold great promise in ameliorating depression among people with alcohol dependency (Shin et al., 2012). If both psychedelics and nature can act as amplifiers for therapeutic effect, this is suggestive that incorporating nature-based settings into psychedelic treatment models could elicit a potential beneficial synergy.

There are many reasons to consider that therapeutic psilocybin sessions, despite the usual ‘inward focus’ could, for some participants, be better supported by being held in a natural setting than in a hospital room. Given that anxiety is a predictor of challenging or anxiety reactions to psychedelics (Haijen et al., 2018; Studerus et al., 2012), the importance of a psychologically soothing setting cannot be overstated. Nature-based settings have a tendency to be inherently more aesthetically pleasing than built environments (Carlson and Berleant, 2004; Richards, 2001; Shafer and Mietz, 1969; Ulrich, 1983) with natural stimuli having the capacity to induce psychologically restorative ‘soft fascination’ (Basu et al., 2018; Stenfors et al., 2019). Such settings have a soothing effect on the mind, allowing for mental space for reflection, with reductions in stress in line with attention restoration theory (the renewal of attention and depleted psychological resources, and reductions in mental fatigue, in a natural environment), in addition to stress reduction theory (Berman et al., 2008; Kaplan, 1995; Kaplan and Kaplan, 1989; Ulrich et al., 1991). Given the capacity of nature contact to reduce rumination and encourage present moment focus (Bratman et al., 2015a, 2015b), holding psychedelic sessions in natural environments could counteract mental preoccupation which has been associated with increased likelihood of challenging experiences with psychedelics (Russ et al., 2019). The soothing effect of a beautiful natural environment may partly explain why they are commonly selected as settings for psychedelic experiences (Kangaslampi et al., 2020; Luke, 2017; Mason et al., 2020; Masters and Houston, 1966; Prepeliczay, 2002; Uthaug et al., 2019) and taking psychedelics with the intent to connect with nature has been associated with greater well-being scores and likelihood of mystical-type experiences in comparison to a number of other potential motivations behind usage (Haijen et al., 2018). Natural settings are also the preferred setting for some indigenous psychedelic using groups, including the Wixáritari (Huichol) of Mexico, the planet’s oldest surviving psychedelic using culture (Lawlor, 2013).

Given that our species has spent almost its entire existence in natural environments, it is likely we have an innate preference for them (Kellert and Wilson, 1995). The perceptual effects associated with psychedelic administration include heightened sensory capacity and altered visual perception (Preller and Vollenweider, 2016; Watts et al., 2017), which can lead to a more absorbing and intensified experience of the environment in which they are taken. When psychedelics are taken in natural/nature-rich settings, the sensory aspects of nature may be perceived more richly and immersively than usual (Krippner and Luke, 2009). For example, a flower may be experienced as overwhelmingly beautiful in its intricacy and vibrancy (Huxley, 1954; Watts et al., 2017). In addition, feelings of interconnectedness with the natural world are likely to be more prominent in outdoor nature-based settings (Cooley et al., 2020; Dutcher et al., 2007; Unsworth et al., 2016; Van Gordon et al., 2018).

However, despite all the benefits of calm, beauty and a sense of interconnectedness with all life that a natural setting could potentially bring, there are major barriers to attempting to hold psychedelic sessions in nature. There may be issues with disturbances, privacy, inclement weather to name but a few (Cooley et al., 2020; Jordan and Marshall, 2010). Indoor settings offer a greater amount of control, comfort and safety than wild outdoor settings.

**Figure 1.**
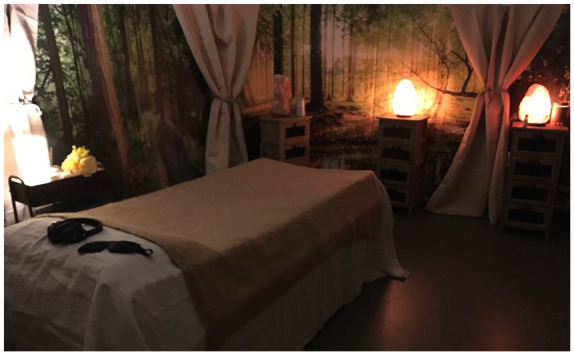
Photo of psilocybin for depression treatment room.

In situations where a clinical room is needed, bringing some natural elements into the clinical space can be beneficial, for example plants (including those that belong to the session participant), nature-based photography and art and a nature-based backdrop. Screens depicting woodland scenes were incorporated into the clinical protocol of the Phase II psilocybin for major depression treatment room at Imperial College London (see Figure 1).

Even better than this would be the use of a hybrid indoor/outdoor secure, sheltered structure incorporating biophilic design elements (Joye, 2007) in a nature-based setting with large skylights and windows. This would allow the therapist to titrate the amount of nature immersion according to the client’s needs as the session progresses. Structures incorporating biophilic design elements, sheltered gardens, based in a rural, nature-based setting will be utilised by the Usona Institute in their future psychedelic therapy treatment centre.

### Potential benefits of natural settings for preparation and integration of psychedelic experiences

Even when psychedelic sessions take place in a clinical environment, elements of nature contact and connection can be incorporated into some aspects of the preparation and integration sessions (before and after the psychedelic session). Preparation and integration sessions for individuals and groups of people undergoing psychedelic therapy could include some elements of nature immersion. The purpose of psychedelic preparation sessions is to establish trust between the person who will be having the psilocybin experience and the therapists who will be supporting them through the psychedelic session. Preparation sessions usually take place the day before, and include psychoeducation about the likely effects of psilocybin, discussion of the participant’s intentions for the upcoming session, and some time for establishing therapeutic rapport (Watts and Luoma, 2020). Horticulture exercises (Scott, 2015) may be a perfect complement to a preparation session; that is, ‘preparing the ground’ for the work to come. Individuals and groups could spend time weeding a patch of land, and tilling the soil, and adding compost as a ritual to mark the cleansing and preparation of the inner landscape (the psyche) ready to receive new insights, and experience psychological growth. Integration sessions, which happen the day after psilocybin sessions, are intended to support the participant in fully understanding any insights discovered during the session, and applying them to their life going forward. Horticultural exercises could be useful here too, that is, planting a seed in the freshly tilled soil, as a ritual to mark a new beginning, and a commitment to provide the psychological conditions for personal growth. Commitments to change may fall flat unless they are ‘fed and watered’ on a daily basis. Participants could take home a seed they have planted in a pot, to care for daily, as they nurture the lessons that are growing within themselves.

Other nature immersion exercises could enhance two other key aspects of preparation and integration sessions: mindfulness training and talking therapy. The practice of Shinrin-Yoku (forest bathing), a Japanese form of nature therapy and active mindfulness practice (Craig et al., 2016; Hansen et al., 2017) contains exercises to assist people to come out of their heads and into their environment, which could be useful at every stage in the psychedelic therapy journey. In the Phase II psilocybin for major depression clinical trial conducted by Imperial College London, mindfulness exercises were used to supplement the preparation and integration of psilocybin for depression treatment. Sitting in a calming sheltered garden (Annerstedt and Währborg, 2011; Cooley et al., 2020) could also enhance mindfulness practice, focusing on the smells of different flowers, the sounds of birdsong and running water, and the soothing appearance of trees or bodies of water. A garden can offer a rich canvas for reflective therapeutic process (Adevi et al., 2018; Adevi and Mårtensson 2013; Corazon et al., 2010, 2012; Kaiser, 1976; Sidenius et al., 2017). Awe walks are a simple intervention where people take a walk with the prior suggestion that they attend to details of the world around them and tap into their sense of wonder. A weekly 15 minute awe walk (over a time frame of 8 weeks) has been demonstrated to increase positive prosocial emotions and facilitate a reduction in mental distress in people’s day to day emotional state over time (Sturm et al., 2020). Awe walks in natural settings post psychedelic experience may be beneficial, helping consolidate any feelings of awe which may persist beyond the psychedelic session (Noorani et al., 2018). Such a practice may also help consolidate feelings of connectedness present post psychedelic session (Carhart-Harris et al., 2018b; Forstmann et al., 2020; Kettner et al., 2019; Noorani et al., 2018; Watts et al., 2017), as an increased acknowledgement of nature has been implicated in increasing connectedness in a broad sense (Passmore and Holder, 2017), and awe and connectedness appear to be strongly linked (Bethelmy and Corraliza, 2019; Nelson-Coffey et al., 2019; van Mulukom et al., 2020).

Parts of both preparation and integration sessions contain standard talking therapy elements: discussion of challenges in life, reflecting on traumatic experiences. Although some participants may feel more comfortable having these discussions with their therapist in a clinical room, others may feel more comfortable ‘walking and talking’ with their therapist in woodlands or gardens adjacent to the clinic (Revell and McLeod, 2016) which could provide a soothing setting for discussions which can be emotionally challenging. Participants often feel anticipatory anxiety during preparation session discussions, and integration sessions often touch on tender places and deep wounds that may have been re-visited in the session. Having such discussions whilst walking and talking in a natural setting could be helpful. Walking barefoot outside has been found to increase nature relatedness (Harvey et al., 2016) and this could also be a deeply grounding exercise for participants and therapists to engage in together. An appropriate safeguard for therapeutic work in nature, which is less contained than a clinical setting, might be that all outdoors activities include more than two people (i.e. a two therapists with a participant, or a group of participants with one or two facilitators). Maintaining standard therapeutic boundaries outdoors may require therapists to engage in some additional training, and some psychoeducation around this may need to be discussed with participants (Cooley et al., 2020).

Another benefit of linking preparation and integration to an outdoor nature-based setting is the possibility of establishing a connection to the outdoors as a ‘therapy room’ one can later return to by themselves to self-sooth (Revell et al., 2014). Establishing a nature habit as part of the integration process may serve to consolidate access to a very helpful resource in an ongoing manner. A nature habit may sustain feelings of connectedness beyond what the psilocybin alone may elicit, especially as the antidepressant effects of psychedelics are rarely permanent. The Synthesis Institute has recently launched a nature-based therapy programme for people undergoing psilocybin therapy for depression, a component of which is individually tailored nature plans, where participants select nature-based hobbies, activities, practices and service options, to encourage a deeper connection to, and more contact with nature for the associated psychological benefits this can foster. It is important to point out that although the restorative potential of nature may be well evidenced, actually going into nature may be very challenging for people suffering from severe depression. Therefore, encouraging contact with nature as a factor which could boost psychedelic integration practices may help people commit to visiting nature even when this feels hopeless or pointless, because such habits may confer such important benefits.

The practice of journaling with an emotional focus has been used to effectively supplement therapeutic psychedelic sessions (Griffiths et al., 2018). Nature journaling (recording three things one enjoys about nature each day for five days) has also been found to increase nature relatedness in a robust and sustained way (Richardson and Sheffield, 2017). This suggests that journaling about nature before and after the psychedelic experience could be incorporated into therapeutic models.

Spending time in nature may be one of the most effective practices for maintaining the benefits of psychedelic sessions. However even if people are not able to access beautiful nature on a regular basis, nature can still teach via metaphor. Two core metaphors offered by nature are of interconnectedness and seasonal change. Interconnectedness – recognition of being a small part of a greater whole, for example, demonstrable in many different ways, and seasonal change – with the recognition that humans, like all of nature, go through cycles of light and dark; death and rebirth can be therapeutically beneficial. Just as nature goes through spring, summer, autumn and winter, so do human beings, and it can be helpful to remember that dark times play their part in the cycle, and that ‘this too shall pass’. Our culture is not synchronised with the rhythms of nature: the valuing of productivity, ‘doing’ and happiness, and devaluing of retreat, sadness and loss, may be a contributing factor in the current mental health crisis. Nature can teach humans how to accept darkness as a fundamental part of life, and one that precedes growth. Recognition of this can be life changing for people who suffer from depression. Metaphors about nature have been incorporated into the ACE model for psilocybin for depression (Watts and Luoma, 2020), an upcoming therapy model for group settings, and an upcoming extended integration model.

### Developing a new model for psychedelic therapy to treat nature disconnection

As well as incorporating nature contact into preparation and integration sessions where the specified intention is to use the psychedelic session for the improvement of mental health, a more specific model of nature-focused psychedelic work could be developed, with the specified intention of enhancement of nature relatedness. In the former context, an internal focus is required, whereas in the latter context a more outwardly focused session could be facilitated. This would add to the array of different therapeutic options in psychedelic work. As therapy models expand and more is learned about optimising the experience, there is a need for a variety of options. Just as with mindfulness meditation practices there are exercises for inner and outer focused mindfulness, this is also applicable to psychedelic usage. Whereas the inner focused clinical session might encourage connectedness to self, the outer focused session in nature could encourage interconnectedness with the environment. There could be a therapeutic model developed which starts with inner-focussed work in an indoor clinical setting before then graduating onto an outdoor nature-based setting once people are more experienced with psychedelic effects.

## Conclusion

Aside from their intrinsically psychologically restorative and soothing qualities, nature-based settings could enhance some aspects of the preparation and integration phases of psychedelic therapy, and could under certain circumstances be used for psychedelic sessions themselves, without any neglect of vital safety concerns regarding safeguarding vulnerable people under the influence of psychedelics. Such settings have the potential to reduce anxiety and rumination, increase mindfulness, and elicit transcendent experiences and feelings of awe and connectedness. Furthermore, given the numerous demonstrated benefits to mental health associated with increasing nature relatedness, maximising its enhancement in combination with psychedelic therapy could constitute an independent and complimentary pathway by which psychedelics can lead to improvements in mental health, with nature contact undervalued and heavily underutilised as a health-promoting resource (Bratman et al., 2019; Maller et al., 2006; Summers and Vivian, 2018).

Future studies should seek to investigate the benefits of natural settings and how they may complement (or supplement) clinical or indoor settings in greater detail, employing fine-grained assessments of the settings in question, with thorough attention to potential risks. In addition, future studies should incorporate nature relatedness measures such as the NR-6 (Nisbet and Zelenski, 2013), or the longer but psychometrically superior Disposition to Connect with Nature scale (Brügger et al., 2011) which may help avoid ceiling issues associated with shorter item measures. Furthermore, the inclusion of introspective attitudinal and behavioural measures of environmental concern, in addition to measures assessing related lifestyle choices and materialistic and consumerist behaviours may enhance the validity of findings and avoid common methods bias (Otto et al., 2018).

The chemist and inventor of LSD and discoverer of psilocybin Albert Hofmann came to view the capacity of psychedelics to reconnect our increasingly nature-alienated species to the natural world as perhaps their most important fundamental property. He recalled that among his most satisfying experiences were hearing people say things like ‘I grew up in the city, but once I first took LSD, I returned to the forest’ (Hofmann et al., 2009: 4).
